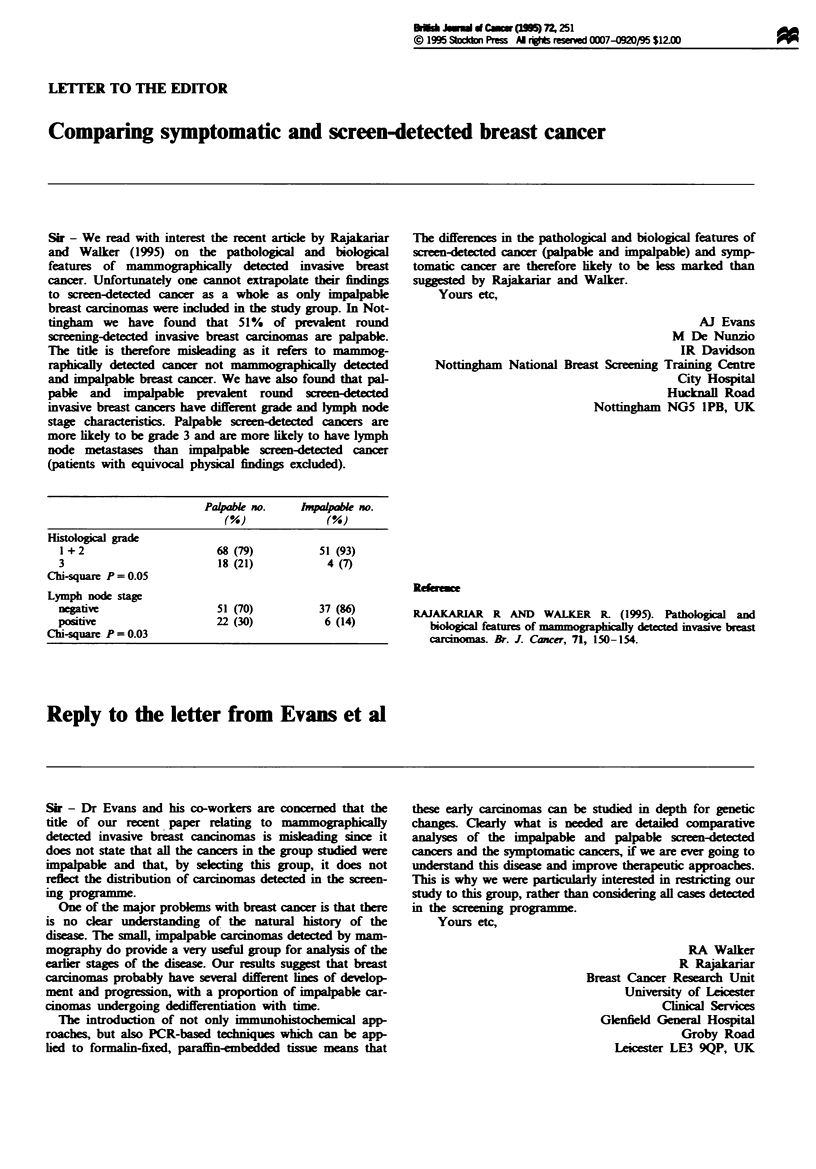# Reply to the letter from Evans et al

**Published:** 1995-07

**Authors:** RA Walker, R Rajakariar


### Reply to the letter from Evans et al

Sir – Dr Evans and his co-workers are concerned that the title of our recent paper relating to mammographically detected invasive breast cancinomas is misleading since it does not state that all the cancers in the group studied were impalpable and that, by selecting this group, it does not reflect the distribution of carcinomas detected in the screening programme.

One of the major problems with breast cancer is that there is no clear understanding of the natural history of the disease. The small, impalpable carcinomas detected by mammography do provide a very useful group for analysis of the earlier stages of the disease. Our results suggest that breast carcinomas probably have several different lines of development and progression, with a proportion of impalpable carcinomas undergoing dedifferentiation with time.

The introduction of not only immunohistochemical approaches, but also PCR-based techniques which can be applied to formalin-fixed, paraffin-embedded tissue means that these early carcinomas can be studied in depth for genetic changes. Clearly what is needed are detailed comparative analyses of the impalpable and palpable screen-detected cancers and the symptomatic cancers, if we are ever going to understand this disease and improve therapeutic approaches. This is why we were particularly interested in restricting our study to this group, rather than considering all cases detected in the screening programme.

Yours etc,

RA Walker
R Rajakariar
Breast Cancer Research Unit
University of Leicester
Clinical Services
Glenfield General Hospital
Groby Road
Leicester LE3 9QP, UK